# Attention-Deficit/Hyperactivity Disorder Predominantly Inattentive Subtype/Presentation: Research Progress and Translational Studies

**DOI:** 10.3390/brainsci10050292

**Published:** 2020-05-14

**Authors:** Ike C. de la Peña, Michael C. Pan, Chau Giang Thai, Tamara Alisso

**Affiliations:** 1 Department of Pharmaceutical and Administrative Sciences, Loma Linda University School of Pharmacy, Loma Linda, CA 92350, USA; cthai@llu.edu (C.G.T.); talisso@llu.edu (T.A.); 2 Department of Psychology, Korea University, Seoul 02841, Korea; petemichael@korea.ac.kr; 3 Division of Social Sciences, University of the Philippines Visayas Tacloban College, Tacloban 6500, Philippines

**Keywords:** ADHD predominantly inattentive, inattention, neuroimaging, comorbidity, genetics, pharmacotherapy, Wistar Kyoto/NCrl, RDoC

## Abstract

Research on the predominantly inattentive attention-deficit/hyperactivity disorder (ADHD-PI) subtype/presentation is important given its high prevalence, but paradoxically it is under-recognized and undertreated. The temporal stability of the inattention symptom could impact the high worldwide prevalence of ADHD-PI. Some evidence suggests differences in the nature of attentional deficit in ADHD-PI vs. that in other subtypes. Impairments in neuropsychological, neurocognitive, and social functioning are also evident in ADHD-PI, which could be specific to the subtype (e.g., processing speed, social perception, and skills), or differ from others in severity. Neuroimaging studies have also revealed ADHD-PI-specific neuropathological abnormalities and those that are shared with other subtypes. ADHD-PI is highly comorbid with learning and internalizing (e.g., anxiety and depression) disorders. There is no solid evidence for ADHD-PI-specific genetic etiologies and differential responses of subtypes to ADHD medications. Translational studies have used the Wistar Kyoto/NCrl substrain which requires further characterizations as an ADHD-PI model. Overall, ADHD-PI research has been conducted in the context of the Diagnostic and Statistical Manual, which arguably does not conform to the widely recognized “dimensional” view of ADHD. The Research Domain Criteria has been proposed to provide a novel framework for understanding the nature of neuropsychiatric illnesses and ultimately improve their diagnosis and treatment.

## 1. Introduction

Attention-deficit/hyperactivity disorder (ADHD) is a highly prevalent, childhood-onset neurodevelopmental disorder which is characterized by developmentally extreme and impairing symptoms that include inattention, motor hyperactivity, and impulsivity, with difficulties that often continuing into adulthood [1,2]. The impairments produced by ADHD cut across multiple domains including educational and vocational problems, familial interaction patterns, and social relationships [3]. As a highly heterogeneous disorder, ADHD manifests differentially across individuals in terms of behaviors, etiology, developmental trajectories, presence of comorbid diagnoses, and response to interventions [3,4]. The heterogeneity in ADHD yields the different subtypes/presentations, namely, predominantly inattentive (ADHD-PI), predominantly hyperactive-impulsive (ADHD-HI), and combined (ADHD-C) presentations (see below).

The terminology and diagnostic criteria for ADHD, similar to other neuropsychiatric disorders, have been improved and developed over the past 50 years, in response to the evolving understanding and conceptualization of the core deficits of the disorder [4,5]. The current diagnostic approach of ADHD utilizes the Diagnostic and Statistical Manual of Mental Disorders (DSM) and its counterpart, the International Classification of Diseases (ICD), which conceptualize ADHD as a categorical diagnosis [4]. This approach, however, has some limitations, necessitating the development and determination of alternative diagnostic structures for ADHD [4].

### 1.1. A Brief History of the ADHD-PI

Since its first appearance in the DSM, there have been controversies surrounding the ADHD-PI subtype of ADHD [5,6]. For instance, the validity of this subtype has been questioned because prior to its representation in the DSM-III in 1980, it was not expected that there would be children who would present significant attention problems without hyperactivity and impulsivity [7]. Since then, a number of studies have examined the validity of this subtype [5,6,7,8]. Moreover, works by Lahey et al. [9,10] and Healey et al. [11] were instrumental in the identification of two ADHD symptom clusters, namely, inattention and hyperactivity/impulsivity, which eventually led to the determination of the three ADHD subtypes in the DSM-IV. As noted earlier, these are the ADHD combined (hyperactivity or impulsivity and inattention present to a significant extent), ADHD-HI (hyperactivity or impulsivity with subthreshold inattention), and the ADHD-PI (significant inattention with subthreshold hyperactivity and impulsivity) types. Table 1 shows the fifth and the latest DSM criteria for ADHD (DSM-5 criteria for ADHD). The DSM-IV criteria for ADHD have been modified, and other notable updates in the DSM-5 include the addition of two ADHD modifiers, and notably, terminological change from ADHD “subtype” to “presentations.”

### 1.2. From “Subtype” to “Presentation”

The DSM-5 criteria for ADHD also reflect a change in the ADHD subtype nosology (i.e., from ADHD “subtypes” in the DSM-IV to “presentations” in DSM-5) in view of the observed instability of ADHD symptoms over time within individuals across their lifespan [12,13]. For instance, many children diagnosed with ADHD-C eventually transition to ADHD-PI, given that the inattention symptom is relatively stable across development. In contrast, the hyperactivity/impulsivity symptoms often diminish with age [14]. Accordingly, the “presentation” terminology better captures the person’s current symptomatology and its instability, in contrast to the more stable, trait-like characteristic denoted by the “type” terminology [15].

Regardless of the current view, the neurobiological underpinnings of the ADHD subtypes/presentations have still been actively explored. Furthermore, a long-standing debate has been whether the subtypes are categorically different disorders that necessitate distinct nomenclature, distinct clinical entities, or conceptually meaningful extremes of the ADHD continuum [4,16,17]. Addressing the above controversy is beyond the scope of this paper. The aim of this review is to discuss the major developments in ADHD-PI subtype/presentation research, highlighting the significant findings obtained from neuropsychology, neuroimaging, genetics, and pharmacotherapy research. It is important to provide up-to-date information on the ADHD-PI subtype/presentation given its high prevalence (see below), the limmited attention it has received over the years, and the temporal stability of the inattention symptom throughout the course of development. Translational research has become increasingly popular and animal models have been used to provide insights into the complex nature of neuropsychiatric disorders in humans. The findings from studies which utilized animal models of ADHD-PI are also described in this review.

## 2. Methods

We reviewed over 100 articles which explored the behavioral and neurocognitive impairments, comorbid features, neurobiology, genetics, and pharmacotherapy of ADHD-PI. The following two strategies were used to identify the articles: systematic search from the two electronic databases (EMBASE and PubMed) and hand search of the reference lists of the included studies. The key terms used for the literature search are listed in Table S1. There was no date limit applied in the search. The final database search was run in April 2020. For studies involving human subjects, an article was included if it met the following criteria: English language studies which assessed individuals with ADHD clinically diagnosed prior to the study and meeting the criteria for ADHD as determined by clinical tools (i.e., standardized ADHD scales), for example, DSM IV (APA, 1994), DSM-IV-TR (APA 2000), DSM-5 (APA, 2013), and ICD-10, rated by clinicians (pediatrician, psychiatrist, or psychologist), teachers, or parents. Articles that included subjects diagnosed with earlier versions of the DSM (DSM-II or DSM-III) were not included due to the fact that they conceptualize and diagnose ADHD subtypes differently than what is currently done in the field. Moreover, ADHD subtypes/presentations need to be clearly identified and used in the analysis of various measures (neuropsychological, neuroimaging, genetic, etc.). Editorials, reviews, commentaries, and case reports were excluded in this review.

During the first stage, any duplicates were discarded, and titles and abstracts were subsequently assessed for their relevance as per the inclusion and exclusion criteria. Papers were rejected outright if (1) they were not published in the English language; (2) were reviews, commentaries, case reports, and editorials; and (3) discussed themes unrelated to or beyond the scope of this study. Two reviewers (M.P. and C.G.T.) separately screened the titles and abstracts for inclusion or exclusion. When consensus on the eligibility of an article could not be achieved, a third reviewer (ID) was involved in the discussion. Full documents were obtained for the remaining records and checked against the eligibility criteria. Papers were rejected at this stage if ADHD-PI was not determined using the DSM or ICD-10, or if ADHD-PI was diagnosed using earlier versions of the DSM (DSM-II or DSM-III). Those which did not report quantitative data were also not included. The papers that met the eligibility requirements were included and discussed in this review. Three authors (I.D., M.P., and T.A.) independently extracted the information from the included studies. The data extracted from the articles, e.g., author, year of publication, study design and setting, clinical tool used, main findings, etc. were listed in a custom data extraction form that conformed with PRISMA guidelines [18].

## 3. Prevalence of ADHD-PI

A comprehensive meta-analysis of 86 ADHD studies in children and adolescents and 11 studies in adults throughout the world reported that the ADHD-PI, as defined by the DSM-IV, was the most prevalent subtype in community samples [19]. Accordingly, the prevalence of ADHD-PI was 23% of preschool children with ADHD which increased further to 45% and 75% of elementary school children and adolescents with ADHD, respectively. Different from ADHD-HI and ADHD-C, the prevalence of ADHD-PI did not decline in the samples of adults with ADHD. In fact, it was the most common ADHD subtype in adults [19]. Nevertheless, individuals with ADHD-PI could have been under-recognized and undertreated given that they were less likely to be referred for clinical services than those with ADHD-C [19]. A 2017 study on ADHD prevalence in a district in China, a region in the world with the largest population of children living in a location with huge diversity in geography and culture, revealed that ADHD-PI was also the most common ADHD presentation. Accordingly, as much as 67.43% of the diagnosed ADHD cases in elementary school students was the ADHD-PI presentation [20]. Furthermore, a recent meta-analysis of ADHD prevalence in Africa also revealed that ADHD-PI was also the most common ADHD subtype, followed by ADHD-HI and ADHD-C [21]. Together, the above studies seem to suggest the unchanging predominance of the ADHD-PI subtype/presentation, in terms of prevalence, in different worldwide scientific studies throughout the years. The consistent nature of this ADHD subtype has been suggested to contribute to its higher incidence [14]. Complementing this, Huang et al. [20] also found that while the hyperactivity/impulsivity symptoms tended to wane early in life, the inattention symptom persisted over time in individuals with ADHD, in line with the above-described temporal stability of the inattention symptom. Meanwhile, there appears to be a gender preponderance of ADHD-PI, in that females are more likely to be diagnosed with this subtype/presentation than males [22,23], although in general, boys with ADHD outnumber girls in clinical settings and population studies [24]. Previous studies have also reported better representation of females over males in the ADHD-PI than in the ADHD-C subtype, particularly in nonreferred community-based samples [25]. Interestingly, the above meta-analysis, in Africa, revealed that ADHD-PI was the most common ADHD subtype in both boys and girls [21]. Future studies should explore the underlying reasons for gender difference in the epidemiology of ADHD in Africa as compared with other countries.

## 4. Behavioral, Neurocognitive, and Neuropsychological Impairments

The symptoms of the ADHD-PI include failing to give close attention to details, difficulty sustaining attention in tasks, not following through on instructions and failure to finish chores or duties in the workplace, difficulty in listening to instructions and organizing tasks, avoiding tasks that require sustained effort, losing things, being easily distracted, and forgetfulness in daily activities (Table 1). While both ADHD-PI and ADHD-C have deficits in the attention domain, clinically significant hyperactive and impulsive behaviors are only present in individuals with ADHD-C. Furthermore, it has been proposed that the nature of the inattention symptom differs between ADHD-PI and ADHD-C. Accordingly, while ADHD-PI is associated with defects in sensory processing and poorly focused attention [26,27,28], ADHD-C is characterized by difficulty in sustaining attention, distractibility, lack of persistence, and disorganization [8,29]. Moreover, children with ADHD-PI sometimes show elevated sluggish cognitive tempo (SCT) symptoms, for example, slow orientation and response to cognitive and social stimuli [26] as compared with children with ADHD-C [8,30,31,32,33].

The inattentive behavior and attention problems are complex and postulated to be related to abnormalities in neurocognitive functions [13,34]. Attention skills can be measured by the continuous performance test (CPT) and its indicators could sensitively identify attentional deficits in ADHD [35]. A meta-analysis that included different CPT versions concluded that one of the most impaired measures in individuals with ADHD is the number of omissions, which indicates a deficit in the ability to respond to target stimuli (i.e., selective attention) [36]. Previous studies have shown that children with ADHD-PI perform worse than the controls and individuals with ADHD-C on the omission measure of the Conner’s CPT [37,38]. Therefore, it has been suggested that the impairment of this ability is highly associated with the inattention criteria of the disorder [37,38]. In another CPT study, ADHD-PI individuals performed worse than typically developing controls not only in measures of selective attention (omissions), but also in other areas of attentional functioning such as the ability to maintain a consistent response time throughout the task, vigilance, processing speed, sustained attention, and inhibitory control [39]. Other ADHD subtypes were not included in the study, and therefore subtype-specific differences could not be determined. Other tests have also been employed to examine inattention in ADHD-PI, and furthermore, to explore whether there are differences in attentional processes in ADHD subtypes. In a counting Stroop task, which measures the mechanisms of cognitive interference while processing number and meaning, and sustained attention, both the ADHD-PI and ADHD-C groups showed greater cognitive interference effect as compared with a healthy control group. This outcome coincides with the above-mentioned deficit in sustained attention and vigilance in individuals with ADHD and suggests similarities in the deficits in interference control and in adopting a consistent response strategy in the two subtypes [40]. In a dichotic listening task which reflects different cognitive processes including attention (forced-right condition), cognitive control (forced left condition), and perception (unforced condition), children and adolescents with ADHD-PI showed significant right-ear advantage also during the forced left condition as compared with ADHD-C and healthy controls. These findings suggest a deficit in cognitive control in ADHD-PI as compared with other subtypes, reflecting the difficulty of individuals with ADHD to inhibit irrelevant stimulus cues in the environment and to refocus on relevant, less salient cues [41].

Abnormalities in executive functions can occur in various neurocognitive functions such as focusing and sustaining attention [42]. While there have been a number of reports that have demonstrated executive function problems in children with ADHD-C, little is known about the executive function deficits in ADHD-PI and whether they are different from other ADHD subtypes/presentations. In a study which utilized teacher ratings of school-related executive function difficulties in seven- to 15-year-old Finnish children, those with ADHD-PI had a wider range of school-related executive function challenges such as planning, initiation, and attention as compared with those with ADHD-C type [43]. In a study of preschoolers (4–5 years old) in China, those with ADHD developed poorly on some aspects of executive functions and related abilities on inhibition, executions, sensorimotor, verbal productivity, and nonverbal reasoning [44]. However, it was found that ADHD-C children had the poorest executive function as evaluated by the Behavior Rating Scale of the Executive Function Preschool Version (BRIEF-P) [44]. The discrepant findings could be explained by the characteristics (e.g., age) of the subjects and the use of different rating scales to assess executive functions. Addressing these methodological considerations, including the sample size limitation, would help rule out possible confounds and establish more conclusive differential dysfunction, or the absence thereof, of subtypes/presentations. Considering that executive functions are also measured using cognition performance-based tests, the use of these types of assessments in future studies would provide further information on the executive function weaknesses of individuals with ADHD-PI.

Neuropsychological processes such as working memory and processing speed are also relevant to the presentation of ADHD symptoms [45]. Working memory is an essential executive function that involves the cognitive capacity to maintain and manipulate information over short periods of time [46]. Deficits in working memory have been observed in children with ADHD regardless of subtype [47,48]. A previous study, however, showed that children with ADHD-PI performed worse on the backward vs. forward tasks, which measured working memory maintenance and manipulation as compared with the children with ADHD-C [49]. It was suggested that different patterns of working memory deficits exist in children with ADHD-PI vs. those with ADHD-C [49]. In addition, processing speed entails the cognitive capacity to process information and engender a correct response within a given time [50]. Previous studies have shown a higher risk of processing speed deficits in children with ADHD-PI relative to typically developing children [51,52] and children with ADHD-C [48]. While adults with ADHD-HI demonstrate faster processing speed [53], those who showed symptoms of inattentiveness displayed slower processing speed [53,54]. The above findings suggest specificity of processing speed impairments in individuals with ADHD-PI. A more recent study also found that processing speed deficits were mostly related to inattention, although not exclusively, rather than the hyperactivity/impulsivity component of ADHD [55]. The authors suggested that the inattention contributed to less efficient performance and worse attention to detail on tasks with a higher perceptual or psychomotor load, whereas the hyperactivity/impulsivity component affected psychomotor speed or incidental learning, possibly via greater inaccuracy or reduced learning efficiency [55].

Despite the fact that many studies reported neuropsychological and neurophysiological differences between ADHD-PI and ADHD-C, it is worth noting that a previous meta-analysis of empirical data revealed findings that did not support neuropsychological distinctions between the two subtypes [8]. It was further suggested that these subtypes differ only in severity [34]. This suggestion, however, did not put an end to the debate on the categorization of the ADHD subtypes/presentations. As mentioned below, some evidence seems to suggest the distinction between the ADHD subtypes. Additionally, another research framework has been proposed to better understand the neurobiology of the symptom domains of ADHD and address the heterogeneity of the disorder (see Section 10).

With regard to social functioning, individuals with ADHD-PI often struggle socially and have problems with social perception. A previous study that compared the social functioning of patients with ADHD-PI and those with ADHD-C found impaired assertiveness in ADHD-PI youths, whereas those with ADHD-C showed deficits in self-control [56]. Differences in social perception were also observed in that those with ADHD-PI were found to perform more poorly than those with ADHD-C on direct measures of social understanding and social skills [57]. Affect recognition abilities were also impaired in adults with ADHD, especially those with ADHD-PI as compared with those with ADHD-C [58]. More recently, Capriotti and Pfiffner [59] reported a lack of social skills and assertion, as well as functional impairments at home and school in youths with ADHD-PI. The social impairment in children with ADHD-PI has long been ascribed to behaviors that are passive and withdrawn, and deficiency in social knowledge, which resulted in neglect and isolation from peers. In contrast, those with ADHD-C are more impulsive and intrusive which leads to active rejection from peers [7,8,56]. In laboratory studies, children with ADHD-PI have particular difficulties with attending to relevant social cues and actively engaging in social interactions [60].

In summary, the accumulating evidence suggests a difference in the nature of attentional deficit in ADHD-PI as compared with other subtypes. Neuropsychological, neurocognitive, and social functioning are also evident in ADHD-PI, which could be specific to the subtype (e.g., processing speed, social perception, and skills), or differ from others only in severity (e.g., executive functions and working memory). In general, the small sample sizes of the above-described studies prevent the generalizability of the findings to the clinical setting. To fully establish ADHD-PI-specific behavioral, neuropsychological, and social functioning impairments, future work should include other ADHD subtypes/presentations (i.e., ADHD-HI) in the assessments. However, recruiting ADHD-HI patients in ADHD studies is a challenge given that they are less frequent in samples as compared with other ADHD subtypes/presentations [61].

## 5. Comorbidity Features

The comorbidity of ADHD with other childhood-onset neurodevelopmental disorders and psychiatric disorders is substantial [2]. While earlier reports showed that co-occurring anxiety disorders or other neuropsychiatric disorders were comparable across ADHD groups [62,63], other studies, including a recent one [64] appeared to indicate that ADHD-PI could have higher vulnerabilities, especially when examined along with other internalizing problems such as withdrawal and depression [8,65]. A study on clinic-admitted Turkish children showed that comorbid anxiety disorder is more common among individuals diagnosed with ADHD-PI subtype, while oppositional defiant disorder (ODD) was significantly more common in ADHD-C type [66]. ODD scores were also higher in boys in the ADHD-C group as compared with the ADHD-PI according to a study of Japanese children [67]. Moreover, it has been found that there was high comorbidity between childhood ADHD, and ADHD-PI, in particular, with social anxiety disorder (SAD) among Turkish patients in an outpatient psychiatry clinic [68]. This was also the finding in a cross-sectional study conducted in Spain which revealed that adolescents with ADHD-PI showed worse social anxiety problems than those presenting with ADHD-HI and ADHD-C [69]. In addition to ODD, the symptoms of hyperactivity and impulsivity were mostly associated with higher rates of comorbidities with aggressive behavior, problematic conduct behaviors, criminal involvement, personal failure, and negative self-statements [8,66,70,71,72,73,74]. A report about clinic-referred adult ADHD patients in Germany found a higher incidence of externalizing (disruptive, hyperactive, and aggressive) behaviors among ADHD-HI and combined types of ADHD [75]. Additionally, a study in Taiwan conducted on ADHD adults also showed that ADHD-PI individuals were less likely to develop persistent ODD and conduct disorders as compared with those diagnosed with ADHD combined type [76].

Regarding substance use, stronger relationships between hyperactive/impulsive symptoms with substance use and abuse/dependence were observed in a number of studies [77]. This is in line with the existing literature reporting that impulsivity is a risk factor of substance use and disorders [78]. A cross-sectional study also found higher gambling problems with those who screened positive as ADHD-C than those with ADHD-PI [79], although both subtypes were equally likely to gamble as compared with non-ADHD controls. Previously, it was found that ADHD-PI children also display some impulsive characteristics (e.g., poor executive response inhibition and effortful control), but their impulsivity was described as an inability to finish tasks when feeling fatigued or bored [80].

Moreover, individuals with ADHD-PI were more likely to have comorbid learning disabilities and long-term academic impairments. In some instances, these learning problems were worse in ADHD-PI as compared with other ADHD subtypes [81,82,83]. Accordingly, reading and mathematics learning disorders have been associated with ADHD-PI and ADHD-C [84]. Furthermore, other studies have revealed a significant association of ADHD-PI with overall reading problems [85,86,87]. For instance, in a clinically referred sample of ADHD children, inattentive behaviors, but not hyperactivity and impulsivity symptoms, were associated with reading fluency and comprehension [87]. Studies conducted in non-clinically referred samples could provide additional information. A more recent study showed that after controlling for fluid intelligence quotient (IQ) level and gender, ADHD-PI children were at higher risk of being in the lowest-performing 10th percentile for reading (three times), writing (>than 3.9 times), and mathematics (>six times) than those with ADHD-C and ADHD-HI [88]. In contrast, significantly higher risks for reading/spelling difficulties and for math difficulties were found in children with high hyperactivity scores [89]. The different outcomes could be explained by a number of factors including teacher vs. parent rating of ADHD symptoms. However, parent- and teacher-reported academic achievements could be influenced by biases, and therefore objective tests are needed to reliably evaluate achievement levels of subjects.

Other comorbidities reported in the literature include disruptions in sleeping patterns such as sleep impairment, sleepiness, and poor sleep functioning [76,90,91,92]. Although it was shown that ADHD subtypes have worse sleep quality or show higher rates of sleep problems [93] as compared with healthy controls [92], some of the studies have reported inconsistent findings with respect to which subtypes have greater sleep dysfunctions [76,91,93]. The controversy could potentially be explained by factors such as gender, treatments, and comorbid psychiatric problems. Nevertheless, it has been found that comorbid anxiety was significantly associated with impaired sleep in individuals with ADHD-PI [90].

Taken together, ADHD-PI is highly comorbid with learning and internalizing (e.g., anxiety and depression) disorders. Further longitudinal and cohort studies to explore the development of these comorbidities across age groups could provide important clinical information about the converging etiologies, as well as in designing potential interventions. It is noteworthy, however, that the overlap between ADHD-PI and anxiety disorders could be a contributing factor to the misdiagnosis or overdiagnosis of ADHD-PI [94,95]. There has been a challenge in the differential diagnosis of ADHD-PI and anxiety disorders, especially with regard to the inattention symptom present in both conditions [95]. Because prior research supports the distinction in the etiology of the inattention symptom in ADHD and anxiety disorders [96,97,98], it is important to develop neuropsychological and diagnostic tools that could better capture the differential profiles for these two disorders.

## 6. Neuroimaging Studies

The advent of neuroimaging studies paved the way for a deeper understanding of the neurobiological bases of ADHD. Moreover, neuroimaging has also been used to explore neuropathological similarities or distinctions between ADHD subtypes/presentations [99,100]. There were only a few studies that examined structural volumetric changes associated with the ADHD subtypes. In contrast to the controls, on the one hand, ADHD-PI children showed smaller volumes of the anterior cingulate cortex, left medial frontal gyri, caudate and thalamus, and right postcentral gyrus gray matter. On the other hand, ADHD-C children displayed a reduction in frontal, parietal, temporal, and occipital lobes relative to the controls [101]. Some studies, however, reported no significant global or specific volumetric differences in the basal ganglia structure among ADHD-PI, ADHD-C, and controls [102,103,104]. In contrast, other studies found bilaterally smaller caudate and anterior cingulate cortex volumes in children with ADHD-C as compared with ADHD-PI and typically developing controls [105]. Considering the small number of studies conducted in this area, more future investigations or replications, with special attention to exploring the roles of other demographic characteristics such as age and gender, could help to clarify these inconsistent findings.

Functional neuroimaging studies, which allow noninvasive measurements of brain functions, have provided further insights into the neurobiology of ADHD-PI and other subtypes/presentations [106]. The groundbreaking work of Solanto et al. [26] provided the first evidence of larger activation in middle frontal, temporal, and parietal regions, in children with ADHD-PI. In contrast, there was greater activation of the bilateral medial occipital lobe in children with the ADHD-C as compared with those with ADHD-PI. To identify a consistent pattern of neural dysfunction in ADHD and ADHD subtypes, a meta-analysis of functional magnetic resonance image (fMRI) studies using Go/no-go, Stop, and N-back tasks were performed [107]. The meta-analysis revealed more profound underactivation in the right superior and inferior frontal gyrus during the Stop task, in the right caudate during the Go/no-go task, and in the right cerebellum during the N-back working memory task, in ADHD-C relative to the ADHD-PI [107]. Areas of the default mode network (DMN), including the medial frontal and occipital regions, were more significantly activated in the ADHD-C subgroup as compared with controls for the above-mentioned tasks [107]. The DMN represents brain areas that are more active during times of rest as compared with times of cognitive activity [108] and has been implicated in regulating goal-directed activity, motivational effort, and attention dysfunctions in ADHD [109]. Dysregulation of the DMN during task performance due to sustained attention deficits has been linked with increased error commission and decreased attentional performance [110,111], and associated with impulsivity symptoms and impaired response inhibition in the ADHD-C subtype [112,113,114].

In an fMRI auditory oddball attention task, which measures attentional orienting and updating of contextual information in working memory [115], ADHD-PI youths showed abnormal neural activity in multiple brain regions, including frontoparietal regions associated with early sensory processing and orienting cognitive processes [116]. These subjects, however, showed little fronto-striatal abnormalities, in stark contrast with ADHD-C adolescents who showed caudate and lateral prefrontal oddball activation deficits [117]. The abnormalities in the parietal areas have been suggested to represent the main difference between the ADHD-PI and ADHD-C subtypes [118]. This is consistent with the assumed involvement of norepinephrine pathways in ADHD [119] and oddball-elicited event-related potentials [120]. Additionally, fronto-striatal deficits have been associated particularly with hyperactive/impulsive ADHD symptoms [116,117]. However, since both ADHD-PI and ADHD-C adolescents showed frontoparietal deficits, it was suggested that these subtypes shared impairment in this neural pathway [117]. Nevertheless, on the one hand, the ADHD-PI adolescents could have shown greater impairment in this region in view of the more profound parietal lobe, insular and cingulate abnormalities in ADHD-PI as compared with ADHD-C [116]. On the other hand, a recent fMRI study showed that adults with ADHD-PI and ADHD-C both showed abnormalities in the fronto-striatal networks during the performance of a counting Stroop task [118]. However, there was more dramatic hypoactivation of the fronto-striato-parietal network in ADHD-C suggesting neurocognitive impairment in this subtype beyond those associated with fronto-striatal networks. It was previously shown that a network of fronto-striato-parietal areas was involved in the control of attention, decision-making, and working memory necessary for successful Stroop performance [121].

Dysfunctions in reward and motivation and susceptibility to drug addiction also characterize ADHD [53,122,123]. In a motivational fMRI paradigm, adults with ADHD-PI showed bilateral ventral striatal deficits during reward anticipation. However, the ADHD-C subjects displayed orbitofrontal hypoactivation to reward feedback [124]. This is an intriguing finding given the negative correlation between hyperactivity/impulsivity and ventral striatal activation during reward anticipation [53,124,125].

Recently, functional and structural connectivity analyses have been employed to understand brain connectivity features of the different ADHD subtypes/presentations. The findings from these studies seemed to suggest distinctive neural networks that were altered in the ADHD presentations [126,127]. Classification analysis of multimodal imaging and phenotypic data associated structural graph theory network measures of the DMN with ADHD-PI as compared with the ADHD-C and ADHD-HI subtypes, and controls [128]. Resting-state functional connectivity MRI (rs-fcMRI) studies that incorporated graph theoretical analysis also reported differential neural activation in the sensorimotor and DMN in ADHD-PI vs. ADHD-C subtypes [112] and ADHD-PI relative to controls [129]. Examining whether the observed functional differences in the DMN correlate with volume and structural covariance within this network is an important area for future investigation. In contrast, there were no functional connectivity differences between ADHD-PI and ADHD-C in drug- naïve adults with ADHD [130]. Furthermore, individuals with ADHD-C and ADHD-PI were distributed evenly along the axis as identified by the canonical correlation analysis, suggesting “dimensional” biotype of childhood-onset adult ADHD (see Section 9) [130].

The adoption of diffusion tensor imaging (DTI) in ADHD studies also provided important information with regard to the structural and white matter connectivity defects in the ADHD subtypes [131,132,133,134]. DTI studies have provided estimations of the directional diffusion of water molecules along axonal pathways and subsequently, the microstructural properties and orientation of the white matter tracts [134,135]. DTI measures, namely mean diffusivity (MD), axial diffusivity (AD), radial diffusivity (RD), and fractional anisotropy (FA) describe the pathological and developmental changes in axonal density and size, degree of myelination, and organizational coherence of fibers within a voxel [135,136]. In particular, the FA values signify the amount of hindrance/restriction experienced by water molecules along the direction of white fiber and have been suggested to represent changes in the white matter microstructure [39,137]. One DTI study showed that children with ADHD-PI have higher FA in the anterior thalamic radiations (ATR), bilateral inferior longitudinal fasciculus (ILF), and in the left corticospinal tract (CST) [131]. The ATR is a fiber bundle that interconnects the frontal cortical regions with the mediodorsal and anterior thalamic nuclei [138], and thus it is an important component of the fronto-striato-thalamic circuits with important implication in executive, social, and motivated behaviors [139]. The ILF provides an associative connection between the temporal region, and abnormalities have been suggested to contribute to the learning difficulties shown by subjects with ADHD-PI [30,140]. Differently, white matter alterations in the CST in an ADHD-PI group have been suggested to correspond to abnormal motor development and an alteration of fine and gross motor skills reported previously in ADHD [141]. In contrast, the ADHD-C group exhibited higher FA in the bilateral cingulum bundle (CB) [131]. The CB is an important area facilitating interactions between DMN hubs [142]. This finding coincides with the above-described alteration in DMN in an ADHD-C subgroup (McCarthy et al., 2014) and has been suggested to underlie executive functions and sustained attention deficits that are present in ADHD-C [140]. Significant abnormalities in radial diffusivity in the ventral portion of corpus callosum (FMi) have been found in ADHD-PI individuals [131]. White matter abnormalities in the FMi have been associated with processing speed [143] that appears to be slower in ADHD-PI individuals than in ADHD-C subjects [144].

A structural connectome study of ADHD-PI and ADHD-C subtypes revealed network organization differences in the context of preserved volume [99]. Significant differences in nodal degree, which estimates the connections that a node has with other neural networks, have been found between ADHD-PI and ADHD-C [99]. In ADHD-PI, the nodal degree was higher in the regions associated with the limbic, visual, and ventral attention network as compared with ADHD-C, consistent with the disrupted frontoparietal and limbic network pathways [99,112,145,146] observed mainly in ADHD-PI [116,117]. The nodal degree in the amygdala was also higher in ADHD-PI as compared with controls, which could subserve poor emotional regulation, social cognition problems that are consistent with the ADHD-PI symptom profile [147]. In ADHD-C, the nodal degree was higher in the cerebellum [99], a brain region associated with the motor network and also known to interact with the frontoparietal executive control circuit [99] as compared with ADHD-PI. Moreover, the nodal degree was higher in the anterior cingulate (a key node of the DMN), middle frontal gyrus, and putamen in ADHD-C as compared with controls, which are related to ADHD-C behaviors of impulsivity, disinhibition and distractibility, goal-directed action, and attentional processing deficits [148]. The lack of cognitive measurements was one of the limitations of this study, and the small sample size, precluded the generalizability of the results [99].

In summary, neuroimaging studies have provided indispensable information to improve our understanding of the neurobiological substrates of ADHD-PI and other ADHD subtypes/presentations. Inadequate sample sizes, methodological heterogeneity, and poor reproducibility remain the big challenges in neuroimaging studies, in general, which could be addressed by collaborative multicenter research [4]. Moreover, the cross-sectional approach employed in most neuroimaging studies does not allow the establishment of causal relationships between neural correlates and ADHD traits. Pharmaco-imaging (combining neuroimaging and randomized controlled trials) is an approach that could circumvent the limitations of correlational neuroimaging research, helps identify causal effects of interventions, and thus is a step toward addressing the issue of causality [149]. The inclusion of female subjects in neuroimaging studies is also ideal given the higher rate of ADHD-PI in females versus males.

## 7. Genetic Studies

Similar to other psychiatric disorders, the pathophysiology of ADHD likely involves the interplay between genetic and environmental risk factors [1]. The genetic influences in ADHD have been reviewed in many studies [1,4,150]. ADHD is a highly heritable condition and its heritability has been estimated to be around 70% and 80% [1,151].

Association studies have been conducted to identify ADHD susceptibility genes including those that are associated with ADHD subtypes. In particular, subtype-specific genetic analyses have been performed with the primary goal of reducing inter-study heterogeneity and, ultimately, finding ADHD candidate genes [152]. Compelling evidence for the involvement of dopaminergic systems in ADHD have resulted in investigations exploring the link between dopamine-related genes, for example, dopamine transporter (*SLC6A3*/*DAT1*), dopamine receptors (e.g., *DRD4* and *DRD5*), and ADHD [151]. A family-based association study of a proband of 273 Chinese children with ADHD, reported variations in the *DAT1* gene, in particular, a haplotype rs27048 (C)/rs429699 (T) to be significantly associated with the inattentive subtype and severity [153]. No allele or haplotype was significantly associated with the hyperactivity/impulsivity severity. Some studies also found stronger *DRD4* polymorphism association with inattention than hyperactive-impulsive symptoms of ADHD [154,155]. Moreover, children with high levels of the endophenotype displayed a stronger association between inattentive symptoms and *DRD4* [154]. In contrast, the *DRD5* has been associated with both ADHD-PI and ADHD-C subtypes [156].

The noradrenergic system, which underscores behavioral activation, alertness, and attention, is another widely explored neurotransmitter system in ADHD pathology. Associations between the *Msp*I polymorphism at the adrenergic α2A receptor (*ADRA2A*) gene and the inattention symptom have been reported in a cohort of Brazilian youths with ADHD [157,158]. Moreover, pharmacogenetic studies have shown greater improvement of inattentive symptoms following one month of methylphenidate treatment in children and adolescents with the G allele (G = G and G = C genotypes) at the *ADRA2A*-1291 C > G polymorphism [159]. Follow-up studies also showed a greater improvement of inattentive symptoms with methylphenidate treatment already in the first month of treatment in children and adolescents with the G allele (G = G and G = C genotypes) at *ADRA2A*-1291 C > G polymorphism in a nonreferred sample of children with ADHD-PI [160]. Aside from the small sample size, there were other limitations of the study [160] that prevented the assumption of a direct relationship between polymorphisms at the *ADRA2A* and ADHD-PI, and improvement of inattention symptoms ascribed to methylphenidate treatment.

Aside from dopamine and norepinephrine, other neurotransmitter systems have also been implicated in ADHD, for instance, serotonergic and cholinergic systems. Genes associated with receptors and transport, as well as synthesis and metabolism (e.g., catechol-O-methyltransferase (*COMT)*, monoamine oxidase (*MAO)*, dopa decarboxylase (*DDC*)) of these neurotransmitters were also explored for their involvement in ADHD-PI. On the one hand, molecular genetic studies, in a Chinese Han population, showed that genes encoding serotonergic receptor *5-HT1B* and cholinergic receptor *CHRNA4* were predominantly associated with ADHD-PI, as well as *COMT*, *MAO-A*, and *DDC* [161,162,163,164,165]. On the other hand, the serotonergic receptor genes *5-HTR2C* and *5-HT1D*, noradrenergic receptor *ADRA2C*, dopaminergic receptor-associated gene *DRD3,* and norepinephrine transporter gene *NET1* were primarily linked with ADHD-C [161,162,163,164,165].

It is important to note that not only neurotransmitter systems are affected in ADHD, but also those that regulate other neural processes and functions [166]. Recently, the association between the disturbance of cerebral asymmetry with the pathogenesis of ADHD has been suggested [167]. Studies showed that the cerebral asymmetry associated gene *BAIAP2*, located on 17q25 and encodes brain-specific angiogenesis inhibitor 1-associated protein 2 (*BAIAP2*), was linked with ADHD in European populations [168]. Moreover, *BAIAP2* was also reported with childhood ADHD in Chinese Han descent, especially for the ADHD-PI [169].

The above-mentioned findings indicate appreciable development in the identification of genetic etiologies of ADHD-PI. This is important progress but cause-and-effect relationships must be established. The contribution of other genes involved in more basic processes (e.g., cell division, adhesion, neuronal migration, synaptic plasticity, extracellular matrix regulation, and cytoskeletal remodeling processes) in the pathology of ADHD-PI must also be investigated [150,170]. In general, the small sample size of genetic studies is one of the reasons for the observed weak associations between specific genetic markers and ADHD traits. Currently, larger-scale, multicenter approaches have been employed to decipher the genetic architecture of ADHD [4,149], which could also uncover ADHD subtype-specific genetic etiologies.

## 8. Pharmacotherapy Studies

Treatment options for ADHD include behavior therapy and medications, for example, widely used stimulants such as methylphenidate and amphetamine, as well as non-stimulants, for example, atomoxetine. Studies that examine the effects of ADHD medications on the different ADHD subtypes/presentations are few in number. The majority of these studies investigated response to psychostimulants, especially methylphenidate. Stein and colleagues [171] reported that 60% of ADHD-PI children responded optimally, as per parents’ and teachers’ ratings, to lower doses of methylphenidate. In contrast, 66%–75% of ADHD-C participants showed optimal improvement of ADHD symptoms to higher doses. Thus, the authors of the study proposed that the improvement in inattentive symptoms was achieved at lower stimulant doses, while higher doses were effective in treating hyperactivity and impulsivity symptoms [171]. However, Solanto et al. [172] reported contrasting findings in that the response to the different doses of methylphenidate for the two ADHD subtypes did not vary significantly. Accordingly, drug effects were predominantly linear for both children with ADHD-C and ADHD-PI. The dissimilar outcomes of the above studies have been attributed to differences in patient characteristics, experimental approaches, the use of parent and teacher rating scales, and other laboratory measures as dependent variables [61]. In addition, in ADHD children who were previously treated with immediate release methylphenidate, the clinical treatment with OROS methylphenidate for 21 days resulted in (parent-rated) symptom control especially in the older age groups (10–16 years old), the higher dose cohort (36 or 54 mg), and those with ADHD-PI [173]. In a recent study [61], it was found that boys with ADHD with high levels of HI (ADHD-HI), showed moderate to significant improvement of ADHD symptoms or associated behaviors in response to methylphenidate, greater than those with ADHD-PI, and consistent with the findings of Stein et al. [171]. However, the above study did not include girls with ADHD and did not examine responses to methylphenidate at different doses. Pharmacological treatment was also combined with behavioral interventions, and thus it was not known to what extent the behavioral interventions influenced the outcomes in the study [61].

The response to methylphenidate in adults with ADHD was also examined. In one of these studies, dexmethylphenidate extended release (ER) was reported to be efficacious in improving ADHD symptoms as compared with a placebo [174]. Analysis of subtype-specific responses did not reveal a significant difference in that dexmethylphenidate ER equally improved ADHD symptoms of patients in the ADHD-PI and ADHD-C categories [174]. This finding coincided with that of a German study which examined the efficacy of methylphenidate treatment in 47 adults with ADHD-PI and ADHD-C [175]. Accordingly, there was no significant difference in the clinical response to methylphenidate in the two groups [175]. However, differences in methylphenidate response were observed between ADHD patients with and without psychiatric comorbidities (e.g., depression). Thus, the ADHD patients who did not show clinically relevant depressive symptoms benefitted more from methylphenidate treatment than those with depression. Furthermore, a study conducted in China showed that methylphenidate improved IQ scores of children with ADHD as compared with untreated controls. However, there was no difference with regard to cognitive improvement as a function of ADHD subtype [176].

The effects of chronic treatment of methylphenidate (initially at 0.5 mg/kg/day and adjusted according to response to treatment) on blood levels of neurosteroids (allopregnanolone and dehydroepiandrosterone (DHEA)) as well as their daily fluctuations in ADHD children were examined in order to discover a potential mechanism underlying the therapeutic effect of this drug. Methylphenidate induced the doubling of allopregnanolone levels in ADHD-PI children without depressive symptoms. The drug did not differently alter DHEA levels in the two ADHD subtypes. The authors of the study concluded that the distinct responses of allopregnanolone and DHEA to methylphenidate suggest the differentiation of effects of pharmacologic manipulations on attention and impulse control. Furthermore, allopregnanolone has been suggested to be a biological marker for ADHD-PI [177]. The same group measured the blood levels of another molecule, the brain-derived neurotrophic factor (BDNF) and its daily fluctuations in ADHD children before and after chronic methylphenidate (initially at 0.5 mg/kg/day) treatment. The authors found decreased serum BDNF in ADHD patients as compared with healthy controls, and importantly, further decreases in serum BDNF with chronic methylphenidate treatment in children with ADHD-PI but not in children with ADHD-C. There have been conflicting findings with regard to the impact of methylphenidate on BDNF levels, and thus the significance of the aforementioned finding remains to be determined [178].

Lisdexamfetamine is a prodrug of D-amphetamine and a widely used ADHD medication. In a group of adults with ADHD, lisdexamfetamine was found to be effective in reducing ADHD symptoms in participants with ADHD-C, ADHD-HI, and ADHD-PI [179]. In line with the above findings on methylphenidate effects on ADHD adults, the clinical response to lisdexamfetamine did not differ significantly by ADHD subtype. A caveat of the study was the inclusion of a small group of participants in the ADHD-HI subgroup in the analysis, which could have impacted the outcomes [179].

Atomoxetine is a nonstimulant approved by the U.S. FDA for use in adults with ADHD. The overall effect size of this drug in ADHD is moderate. Moreover, large, placebo-controlled trials of atomoxetine treatment in adults and young adults with ADHD, adults with ADHD comorbid social anxiety disorder, or alcohol substance use disorders revealed similar extent of ADHD symptom alleviation in adults with ADHD-PI or ADHD-HI [180]. The response to another non-stimulant medication, guanfacine, was examined in a large sample of youths (ages 6–17 years old) with ADHD-PI, ADHD-C, and ADHD-HI [181]. In general, guanfacine reduced ADHD symptoms across ADHD subtypes. Youths with ADHD-C showed greater placebo-adjusted improvements from a baseline in all points than that of the ADHD-PI subjects, although this effect has been ascribed to differences in the baseline symptoms of the two groups [181].

Overall, the responses to stimulant and non-stimulant ADHD treatments do not seem to vary across ADHD subtypes/presentations [182,183,184]. Interestingly, a controlled trial of metadoxine reported that it was well-tolerated and efficacious in treating ADHD symptoms in adults, and that the drug was much more efficacious in improving symptoms and quality of life of subjects with ADHD-PI than those with ADHD-HI [185]. Metadoxine is a non-stimulant drug currently investigated as a potential ADHD drug [185]. The subtype-specific effects of metadoxine await replication in subsequent studies with much larger samples, especially in children and youths with ADHD. The inclusion of females with ADHD-PI in future pharmacotherapy studies is crucial not only due to the high prevalence of ADHP-PI in females, but also because of the observation that girls with ADHD-PI experience more severe academic and social impairment than boys with ADHD-PI [186]. It would also be interesting to examine the efficacy of behavioral treatments in conjunction with pharmacological therapies in order to assess the clinical utility of multimodal treatments of ADHD-PI.

## 9. Animal Models for Translational Research

Animal models of human diseases are commonly used to gain insights into the pathogenesis of disorders and to disentangle the effects of therapeutic regimens. Although animal models cannot truly mimic all aspects of human psychiatric disorders, they can provide insights into disorders that are impossible to be obtained from human studies due to some limitations [187]. In particular, ADHD studies have benefitted from studies in animal models, which provide a well-controlled experimental setup to characterize neurobiology, the effects of neuropathology, and brain effects of chronic drug treatment [187,188].

Sagvolden et al. were the first to propose an animal model of ADHD-PI, i.e., the adolescent Wistar Kyoto WKY/NCrl substrain (obtained from Charles River, Germany), provided that the Wistar Kyoto rat, or the WKY/NHsd (WKY obtained from Harlan, UK) was used as a control (for review see [29]). The importance of specifying the subline codes and country of origin of animals was suggested in order to eliminate discrepancies in results and interpretations in studies involving the ADHD animal models that their group proposed [29]. According to previous studies, the WKY/NCrl was substantially genetically diverse from the WKY/NHsd; and some of the genes that were upregulated in the WKY/NCrl relative to WKY/NHsd include tyrosine hydroxylase (*TH*), *DAT1,* and the solute carrier family 9 (sodium/hydrogen exchanger) member 9 (*SLC9a9*, or *NHE9*) [189,190]. Behavioral studies found that the WKY/NCrl showed inattention without overactivity or impulsiveness in the visual discrimination task as compared with the WKY/NHsd [191]. In contrast, the spontaneously hypertensive rat (SHR)/NCrl substrain (obtained from Charles River, Germany) showed all ADHD-like behaviors [191]. Further characterizations of the SHR/NCrl by their group and others led its recognition as the most appropriate and best validated animal model of ADHD-C, as compared with the WKY/NHsd (for review see [29]). There are also several studies which showed inattentive-like behavior in the WKY/NCrl and SHR/NCrl strains relative to other control strains, for example, outbred Sprague Dawley (SD) and Wistar/HanTac strains (obtained from Taconic Europe) [192,193,194]. However, the use of the SD and Wistar/HanTac rats as controls has been discouraged because of their substantial genetic or behavioral differences from the WKY/NHsd [29,195]. Nevertheless, the use of those strains could potentially circumvent the confusion caused by the employment of the WKY as a control strain [196] or represent the “normal” heterogeneous population [197,198]. In our studies, we found that the WKY/NCrl showed inattention-like behavior and were much more “inattentive” than the SHR/NCrl [197] in the Y-maze task, which measures spontaneous alternation behavior that requires attention. Overall, the WKY/NCrl and SHR/NCrl showed inattentiveness as compared with outbred Wistar rats (obtained from Charles River). Of course, there are limitations to the above behavioral tools to measure attention in animals [199]. Attention is not a single and well-localized function but represents a multidimensional construct [200]. Future studies are warranted to prove inattention-like behavior of WKY/NCrl using other more sophisticated behavioral paradigms (e.g., five-choice serial reaction time task, CPT) [201] and to demonstrate its veracity as an animal model of ADHD-PI. In these prospective studies, appropriate controls strains of the WKY/NCrl ADHD-PI model, including strains that represent the normal, “heterogeneous” population should be utilized for more accurate interpretations and reproducibility of the results.

In view of the proposed role of dopamine in ADHD, Roessner et al. [195] examined dopaminergic neurotransmission in WKY/NCrl. Using quantitative real-time polymerase chain reaction tests, they found that the *DAT* and *TH* gene expression was in the substantia nigra (SN) or ventral tegmental area (VTA) of WKY/NCrl and SHR/NCrl were increased as compared with the WKY/NHsd, indicating that dopamine synthesis and reuptake are elevated in these two strains as compared with a control. However, different from the SHR/NCrl, the breakdown of dopamine was not accelerated and the excitatory DRD1 drive to the SN/VTA was not increased in WKY/NCrl [195]. Using ligand binding assays, they also found that the striatal DAT density was increased in both strains, however, there were greater elevations in SHR/NCrl as compared with the WHY/NCrl. Due to the fact that elevated DAT density has been associated with impulsive behaviors [202], which is present in ADHD-C patients but to a lesser degree in ADHD-PI, the above findings could explain the behavioral differences between WKY/NCrl and SHR/NCrl. Furthermore, Roessner et al. [195] found decreased striatal DAT binding with increasing age in WKY/NCrl and SHR/NCrl (however, there were different developmental courses of striatal DAT density change in WKY/NCrl and SHR/NCrl), which could explain, in part, why the symptoms of hyperactivity/impulsivity tend to wane with age [14,195]. The study of Miller et al. [203] also showed differences in the WKY/NCrl and SHR/NCrl in regulating dopamine release and uptake in the striatum and nucleus accumbens as compared with each other and with the control WKY/NHsd and SD strains. Accordingly, the WKY/NCrl showed faster dopamine uptake in the nucleus accumbens versus the SD control, whereas the SHR/NCrl had faster dopamine uptake in the ventral striatum and nucleus accumbens versus the WKY/NHsd and SD strains [203]. These differences, although subject to further verification, could underlie the above-described behavioral differences between the two ADHD animal models relative to controls.

As noted earlier, Franke et al. erstwhile suggested the involvement of more basic neuronal processes in ADHD [150,170]. We found that in comparison with the Wistar control strain, the WKY/NCrl and SHR/NCrl also showed alterations in prefrontal cortical expression of genes involved in transcription (e.g., *Creg1, Thrsp,* and *Zeb2*), synaptic transmission (e.g., *Atp2b2, Syt12,* and *Chrna5*), neurological system process (e.g., *Atg7, Cacnb4,* and *Grin3a*), and immune response (e.g., *Atg7, Ip6k2,* and *Mx2*) [197]. Our recent studies showed that overexpression of the thyroid hormone-responsive (*Thrsp*) gene in the striatum of mice produced inattention as measured by the novel object recognition and the Y-maze tasks [204]. The expression of dopamine-related genes in the striatum of these animals was also significantly altered [204]. More studies are needed to determine the translational value of these findings in ADHD-PI studies.

As noted earlier, differences in inattention profiles between individuals with ADHD-C and ADHD-PI have been proposed. Recent studies showed differences in stimulus control between WKY/NCrl and SHR/NCrl, which was suggested to support the above proposal [205]. In particular, WKY/NCrl behavior was not under the control of cue lights akin to the passive, orientation, alertness, and sensory processing deficits associated with ADHD-PI in humans [205].

The above-described findings point to the promise and potentiality of the WKY/NCrl as an animal model of ADHD-PI. In addition to further behavioral, genetic, and neurobiological characterizations, the effects of ADHD treatments should be investigated in this model to verify its predictive validity. Moreover, to satisfy face validity, female WKY/NCrl animals should also be included in future studies. It is notable that the WKY/NCrl has also been used as an animal model of depression and anxiety-related behaviors [206,207]. Therefore, the neurobiological correlates of the comorbidity between anxiety and depression and ADHD-PI could also be potentially studied in this animal model.

## 10. Research Domain Criteria to Explore Neurobiology of ADHD and ADHD Presentations

As noted earlier, the DSM-5 and ICD-11 conceptualize ADHD as a categorical diagnosis, which has been criticized because of the widely supported “dimensional” view of ADHD. Accordingly, ADHD is best understood as the extreme end of a continuum, and that ADHD patients differ from those without ADHD by “degree” rather than in kind [4]. Due to the limitation in the current diagnostic approach, a new research framework, the Research Domain Criteria (RDoC) of the USA-based National Institute of Mental Health has been instituted [208]. Among the goals of the RDoC are to understand the nature of mental health and illness in terms of varying degrees of dysfunctions in general psychological/biological systems [209], and to identify the specific neural circuitry underlying typical and atypical behaviors and symptoms in order to improve the diagnosis, treatment, prevention, and cure of mental disorders [208,210]. The RDoC initially associates signs and symptoms regardless of diagnosis, and even including healthy controls, with endophenotypes to further identify etiologies [211]. Thus, as per the RDoC, the individual symptoms of ADHD could have different underlying causes and mechanisms, yet these neurobiological underpinnings resemble those seen in other neurodevelopmental/neuropsychiatric disorders presenting these symptoms, or during a typical developmental period (e.g., executive dysfunctions in ADHD and autism spectrum disorders, or hyperactivity/impulsivity in ADHD and typically developing children) [212,213]. Moreover, ADHD subtypes may not be subtypes, but rather that the individual symptoms of ADHD have different neurobiological underpinnings and mechanisms. Indeed, fascinating findings in genomics have shown shared genetic vulnerability across different types of major psychiatric disorders [214]. Furthermore, the RDoC assumes that the same treatment could be utilized to target the same symptom present in different conditions [213]. The RDoC framework has been applied to research relevant to ADHD and emerging work is beginning to evaluate its relevance to related behavioral manifestations such as conduct problems [215], as well as working memory and reward processing deficits [216]. It is currently bringing promising results, however, the advantages and disadvantages of the RDoC would have to be known in the upcoming years.

## 11. Conclusions

As a prevalent but under-recognized ADHD subtype/presentation and given the temporal stability of the inattention symptom as compared with other ADHD symptoms, the ADHD-PI and the neurobiological underpinnings of the inattention symptom warrant further investigation. There has been some progress, but much remains to be done in order to obtain a more thorough understanding of the nature of ADHD-PI and the inattention symptom. Indeed, some significant strides were found in some areas, for example, neuroimaging research, but the clinical implications of the findings from these studies are hampered by the inherent limitations of the approach, the small sample size in these studies, etc. [4]. With increasing acceptance and support for the dimensional view of ADHD, a clarion call has been sounded to move past traditional approaches and adopt new strategies to study the underlying pathophysiology of psychiatric disorders, as exemplified by the RDoC framework. There have been some advancements, but also challenges with the application of this strategy to explore the etiology and pathophysiology of ADHD [4,215,216,217,218]. Furthermore, the integration of RDoC with other approaches, such as the developmental psychopathology, has been proposed to better parse the developmental divergence of ADHD symptoms and their determinants [216].

Translational research on ADHD-PI is still in its infancy. The WKY/NCrl is not a perfect ADHD-PI model and more characterizations are required to verify that it fulfills the requirements of a reliable and valid animal model of psychiatric disorders [219]. Modeling accurately the different ADHD presentations in animals is crucial for understanding the neurobiology of ADHD presentations and in the preclinical investigations of subtype-specific treatments. However, this could pose some challenges, especially when viewed in the context of the RDoC system, given the complexity of the cognitive domain and the other developmental programs and psychosocial influences of human disorders [220].

## Figures and Tables

**Table 1:** DSM-5 Criteria for Attention-deficit/hyperactivity disorder (ADHD).

Criterion	Description
A	A persistent pattern of inattention and/or hyperactivity-impulsivity that interferes with functioning or development, as characterized by (1) and/or (2):
	**Inattention:** Six (or more) of the following symptoms have persisted for at least 6 months to a degree that is inconsistent with developmental level and that negatively impacts directly on social and academic/occupational activities: *Note: The symptoms are not solely a manifestation of oppositional behavior, defiance, hostility, or failure to understand tasks or instructions. For older adolescents and adults (age 17 and older), at least five symptoms are required.* Often fails to give close attention to details or makes careless mistakes in schoolwork, at work, or during other activities (e.g., overlooks or misses details, work is inaccurate). Often has difficulty sustaining attention in tasks or play activities (e.g., has difficulty remaining focused during lectures, conversations, or lengthy reading). Often does not seem to listen when spoken to directly (e.g., mind seems elsewhere, even in the absence of any obvious distraction). Often does not follow through on instructions and fails to finish schoolwork, chores, or duties in the workplace (e.g., starts tasks but quickly loses focus and is easily sidetracked). Often has difficulty organizing tasks and activities (e.g., difficulty managing sequential tasks; difficulty keeping materials and belongings in order; messy, disorganized work; has poor time management; fails to meet deadlines). Often avoids, dislikes, or is reluctant to engage in tasks that require sustained mental effort (e.g., schoolwork or homework; for older adolescents and adults, preparing reports, completing forms, reviewing lengthy papers). Often loses things necessary for tasks or activities (e.g., school materials, pencils, books, tools, wallets, keys, paperwork, eyeglasses, mobile telephones). Is often easily distracted by extraneous stimuli (for older adolescents and adults, may include unrelated thoughts). Is often forgetful in daily activities (e.g., doing chores, running errands; for older adolescents and adults, returning calls, paying bills, keeping appointments).
	2. **Hyperactivity and Impulsivity:** Six (or more) of the following symptoms have persisted for at least 6 months to a degree that is inconsistent with developmental level and that negatively impacts directly on social and academic/occupational activities: *Note: The symptoms are not solely a manifestation of oppositional behavior, defiance, hostility, or a failure to understand tasks or instructions. For older adolescents and adults (age 17 and older), at least five symptoms are required.* Often fidgets with or taps hands or feet or squirms in seat. Often leaves seat in situations when remaining seated is expected (e.g., leaves his or her place in the classroom, in the office or other workplace, or in other situations that require remaining in place). Often runs about or climbs in situations where it is inappropriate. (Note: In adolescents or adults, may be limited to feeling restless.) Often unable to play or engage in leisure activities quietly. Is often “on the go,” acting as if “driven by a motor” (e.g., is unable to be or uncomfortable being still for extended time, as in restaurants, meetings; may be experienced by others as being restless or difficult to keep up with). Often talks excessively. Often blurts out an answer before a question has been completed (e.g., completes people’s sentences; cannot wait for turn in conversation). Often has difficulty waiting his or her turn (e.g., while waiting in line). Often interrupts or intrudes on others (e.g., butts into conversations, games, or activities; may start using other people’s things without asking or receiving permission; for adolescents and adults, may intrude into or take over what others are doing).
B	Several inattentive or hyperactive-impulsive symptoms were present prior to age 12 years.
C	Several inattentive or hyperactive-impulsive symptoms are present in two or more settings (e.g., at home, school, or work; with friends or relatives; in other activities).
D	There is clear evidence that the symptoms interfere with, or reduce the quality of, social, academic, or occupational functioning.
E	The symptoms do not occur exclusively during the course of schizophrenia or another psychotic disorder and are not better explained by another mental disorder (e.g., mood disorder, anxiety disorder, dissociative disorder, personality disorder, substance intoxication or withdrawal).
***Specify whether:***	
**Combined presentation:** If both Criterion A1 (inattention) and Criterion A2 (hyperactivity-impulsivity) are met for the past 6 months. **Predominantly inattentive presentation:** If Criterion A1 (inattention) is met but Criterion A2 (hyperactivity-impulsivity) is not met for the past 6 months. **Predominantly hyperactive/impulsive presentation:** If Criterion A2 (hyperactivity-impulsivity) is met but Criterion A1 (inattention) is not met over the past 6 months.	
***Specify if:***	
**In partial remission:** When full criteria were previously met, fewer than the full criteria have been met for the past 6 months, and the symptoms still result in impairment in social, academic, or occupational functioning.	
***Specify current severity:***	
**Mild**: Few, if any, symptoms in excess of those required to make the diagnosis are present, and symptoms result in only minor functional impairments. **Moderate**: Symptoms or functional impairment between “mild” and “severe” are present. **Severe**: Many symptoms in excess of those required to make the diagnosis, or several symptoms that are particularly severe, are present, or the symptoms result in marked impairment in social or occupational functioning.	

Note: American Psychiatric Association. Diagnostic and Statistical Manual of Mental Disorders, Fifth Edition (Copyright © 2013).

## Supplementary Materials

The following are available online at https://www.mdpi.com/2076-3425/10/5/292/s1, Table S1: Systematic search process and terms for study questions.
