# Supplementary material for: Attention-Deficit/Hyperactivity Disorder Predominantly Inattentive Subtype/Presentation: Research Progress and Translational Studies
Source: Brain Sci. 2020 May 14;10(5):292. doi: 10.3390/brainsci10050292 (PMC7287898; doi:10.3390/brainsci10050292)
Supplement: Supplementary file 1 [file brainsci-10-00292-s001.pdf]

Supplementary Table S1: Systematic search process and terms for study questions

Question	Search process (Key terms in Pubmed and Embase)	Results
**What is known about the behavioral impairments in ADHD-PI?**	*attention deficit hyperactivity disorder predominantly inattentive AND behavioral impairment*<br>*attention deficit hyperactivity disorder predominantly inattentive AND behavioral problem*<br>*attention deficit hyperactivity disorder predominantly inattentive AND behavioral deficit*<br>*attention deficit hyperactivity disorder predominantly inattentive AND inattention*	# of articles after literature search: **375**<br># of duplicates: **198**<br># of studies met the inclusion criteria: **65**
**What is known about the neurocognitive impairments in ADHD-PI?**	*attention deficit hyperactivity disorder predominantly inattentive AND neurocognitive impairment*<br>*attention deficit hyperactivity disorder predominantly inattentive AND neurocognitive dysfunction*<br>*attention deficit hyperactivity disorder predominantly inattentive AND neurocognitive deficit*	# of articles after literature search: **31**<br># of duplicates: **16**<br># of studies met the inclusion criteria: **10**
**What are the comorbidity disorders or features of ADHD-PI?**	*attention deficit hyperactivity disorder predominantly inattentive AND comorbid features*<br>*attention deficit hyperactivity disorder predominantly inattentive AND comorbid disorders*	# of articles after literature search: **169**<br># of duplicates: **45**<br># of studies met the inclusion criteria: **18**
**What is known about the neurobiology of ADHD-PI?**	*attention deficit hyperactivity disorder predominantly inattentive AND neuroimaging*<br>*attention deficit hyperactivity disorder predominantly inattentive AND neuroanatomy*	# of articles after literature search: 6**2**<br># of duplicates: **36**<br># of studies met the inclusion criteria: **12**

	attention deficit hyperactivity disorder predominantly inattentive AND structural volume<br>*attention deficit hyperactivity disorder predominantly inattentive AND functional neuroimaging*<br>*attention deficit hyperactivity disorder predominantly inattentive AND structural connectome*	
**What is known about the genetics of ADHD-PI?**	*attention deficit hyperactivity disorder predominantly inattentive AND genetics*<br>*attention deficit hyperactivity disorder predominantly inattentive AND genes*	# of articles after literature search: **83**<br># of duplicates: **37**<br># of studies met the inclusion criteria: **9**
**How do ADHD-PI patients respond to medications ?**	*attention deficit hyperactivity disorder predominantly inattentive AND pharmacotherapy*<br>*attention deficit hyperactivity disorder predominantly inattentive AND methylphenidate*<br>*attention deficit hyperactivity disorder predominantly inattentive AND atomoxetine*<br>*attention deficit hyperactivity disorder predominantly inattentive AND amphetamine*	# of articles after literature search: **214**<br># of duplicates: **78**<br># of studies met the inclusion criteria: **15**
**What are the findings of animal research on ADHD-PI ?**	*attention deficit hyperactivity disorder predominantly inattentive AND animal models*<br>*attention deficit hyperactivity disorder predominantly inattentive AND Wistar Kyoto rat*	# of articles after literature search: **24**<br># of duplicates: **13**<br># of studies included in the review: **4**
